# Cement Waste During Primary Total Knee Arthroplasty and its Effect on Cost Savings: An Institutional Analysis

**DOI:** 10.7759/cureus.3637

**Published:** 2018-11-26

**Authors:** James R Yan, Stephan Oreskovich, Kayode Oduwole, Nolan Horner, Vickas Khanna, Anthony Adili

**Affiliations:** 1 Orthopaedics, McMaster University, Hamilton, CAN

**Keywords:** cost evaluation, arthroplasty, health economics, waste, knee

## Abstract

Purpose

Healthcare costs are increasingly garnering more media attention and there is increasing focus on improving efficiencies in daily practice. Orthopedic surgery is also subject to these fiscal pressures, particularly in arthroplasty surgeries, secondary to high volumes with costly equipment. Total knee arthroplasties (TKA) are one of the most common surgical procedures, with over 64,000 annual cases in Canada. Even marginal cost reductions per procedure can be compounded over the large volume to result in considerable savings. This study’s purpose is to investigate and quantify the cost of wasted intraoperative cement used in primary TKA.

Methods

Residual amounts of wasted bone cement were collected and measured following uncomplicated primary TKAs performed by the senior authors in a high-volume arthroplasty centre between January and June 2017. Stryker Simplex® with Tobramycin Bone Cement was the specific institutional cement used.

Results

One hundred and two primary total knee arthroplasties were investigated. The results revealed that an average 91.2 g of surgical cement was wasted per case, with less than 30 g retained in the bone-implant interface (26.8 g). Institutional costs per package of cement is $120.62, amounting to $2.04 per gram of cement. This represents a value of $186.25 CAD per case.

Conclusion

On average, each primary TKA procedure wastes 91.2 g of bone cement per case. The value of this wasted cement is $186.25 CAD per TKA. When extrapolated to the most recent recorded numbers of TKAs done in Canada, that figure nears $12 million. The results of this study are important, as they reveal a potential source to target for both waste reduction and cost control.

## Introduction

The rising prevalence and cost of joint replacement surgery is becoming increasingly burdensome on healthcare institutions throughout Canada. A recent report from the Canadian Joint Replacement Registry estimated 53,207 hip arthroplasties and 64,204 knee arthroplasties in 2016, an 18.1% and 15.7% increase respectively, since 2011 [1]. Primary knee replacements, or total knee arthroplasty (TKA), now comprise over half of all total joint arthroplasty procedures, which, according to North American projections, is a trend with discernible momentum [1, 2]. With the total estimated cost of a single TKA amounting to $10,000 CAD, jurisdictions and taxpayers alike will be expected to absorb potentially billions of dollars in heightened economic demand [3]. As such, there is palpable pressure on hospitals and orthopaedic surgeons to integrate cost control into modern practice.

In 2014, hospitals comprised 29.5% of Canada’s total health spending, and approximately 6% of this budget was consumed by surgical and operating costs [4, 5]. Surgeons are specifically encouraged to augment the cost-efficiency of their practices by adopting standardized evidence-based care through clinical pathways, using new and economic component designs, establishing sustainable supply management pathways, and being conscientious of the waste created during operations [6-9]. The latter notion is at the forefront of recent consideration as operating room waste can contribute up to 70% of a hospital’s total ecological footprint [9]. A surgical waste audit of five TKAs, which was conducted at an Ontario hospital, attributed prosthetics and implants to 17% of the total waste production, and reported an alarming 55.9 kg of non-recyclable waste and prepared but unused materials (i.e., “overage”) [10]. These trends occur throughout North America, as total waste from a single TKA performed in the United States can be valued between $516 and $4216 USD [11].

Given that there are many sources of waste from a single arthroplasty case, investigators have explored various avenues of waste management such as replacing blue polypropylene instrument wrap with recyclable hard cases [12], minimizing the amount of “overage” [13], and repurposing used devices [14]. Another costly, modifiable, and yet often overlooked component of TKAs is intraoperative cement (polymethylmethacrylate) use. In our institution, cement is provided in 40 g bags of polymethylmethacrylate cement powder to be mixed with 20 mL of polymer liquid (Stryker, Mahwah, New Jersey). Standard practice for our institutions’ arthroplasty surgeons is to open and mix two packets of this cement per TKA. However, much of the prepared cement is not needed once the bone and implant interface is established, and therefore it is scraped away from the surgical field and ultimately discarded. Maheshwari et al. have challenged this notion of the necessity for two packets by using only one packet and observing no difference in clinical outcomes or implant survivorship after three years of follow-up [15].

To date, a number of studies [10-12] have looked into arthroplasty cases as contributors to operating room waste [16]. Considering the potential savings associated with reduced intraoperative cement use [15], the authors felt that a pragmatic analysis of cement waste during a standard TKA procedure would be reasonable to consider. Therefore, the primary objective of this cross-sectional and prospective assessment study is to quantify the cost of wasted intraoperative cement for a TKA. The secondary objective is to identify whether or not cement waste correlates to component size. Overall, we expect the results of this study to establish the economic impact of intraoperative cement waste during TKA.

## Materials and methods

Our study obtained approval from our institutional ethics review board. Over a period of six months (January to June 2017), we prospectively recruited 102 patients undergoing primary TKA within a single institution. All surgeries were performed at this institution by two arthroplasty fellowship trained surgeons. Only patients with a preoperative diagnosis of osteoarthritis or rheumatoid arthritis were included. Those who underwent revision surgery, unicompartmental arthroplasty, an additional procedure, previous realignment surgery, or with a complex medical condition requiring additional instrumentation or technical changes from the surgeon’s usual surgical routine were excluded from the present analysis.

Surgical and cementing technique

A Stryker Triathlon® Total Knee System was used by both surgeons for all reported cases. To further ensure standardization among all surgeries, the same two operating rooms were used throughout, instrument sets were kept consistent, a standard TKA lay out was implemented, and a standardized surgical technique using medial parapatellar approach was performed. Intraoperative judgments were made on a case-by-case basis by the main operating surgeon, which included the selection of a Cruciate-Retaining or Posterior-Stabilized implant, and the decision to proceed with patellar resurfacing.

Following all bone cuts, sizing, and implant trialing, polymethylmethacrylate cement was mixed while the wound was thoroughly irrigated with pulsatile lavage method. A Stryker Simplex® with Tobramycin Bone Cement, and Advanced Cement Mixing System were used to mix the cement under vacuum conditions. The staff surgeon would then apply the cement using a typical pressurized cement gun to the cut bone surfaces and the underside of the bone implants. As implant components were fitted onto the bone surfaces and impacted, any excess cement extruding from the component-bone interface was meticulously removed, collected, and ultimately weighed. Any unused cement remaining in the application gun and vacuum mixing system was also accounted for by recording their differences in weight before and after each procedure. All measurements were taken using a digital scale (Trudeau Corporation, Boucherville, Quebec). We calibrated the scale using a standardized 50 g calibration weight.

Data collection and analysis

We prospectively gathered basic patient demographic information (age, gender, operated limb side, and body mass index (BMI)), as well as component sizes. All patient demographic, clinical, and operative information was then compiled into a private spreadsheet and exported for analysis. We used descriptive statistics and reported the data by means with standard deviations or by using medians and ranges. Multiple linear regression analysis was performed to determine if there was a correlation between femoral implant size, tibial implant size or whether or not the patella was resurfaced on total cement waste.

## Results

During the study period, a total of 102 patients were prospectively recruited (56 females, 46 males, mean age 67.9 ± SD 9.0 years). Overall, 46 left and 56 right TKAs were performed by two surgeons. Further demographic data is summarized in Table 1.

**Table 1:** Demographic data from patients in study. Age, height, weight, operative knee, and gender were collected prior to the operation. SD: Standard deviation; BMI: Body mass index; TKA: Total knee arthroplasty.

	Count	Mean (SD)
Number of patients	102	-
Gender (M:F)	46:56	-
Age (y)	-	67.9 (9.0)
Weight (kg)	-	93.6 (19.4)
Height (cm)	-	165.7 (10.7)
BMI (kg/m^2^)	-	34.2 (6.9)
Right TKAs	56	-
Left TKAs	46	-

To ensure accurate quantification of all the potential sources of cement waste during surgery, the weight of all cement-containing instruments on their own was accounted for. To summarize, the empty cement gun tube and the empty cement mixer were 87 g and 315 g, respectively. In terms of the cement itself, the weight of each standard Simplex® with Tobramycin package totaled 59 g when powder and liquid polymer were combined and allowed to set on its own. Therefore, a total of 118 g of cement was available during a standard procedure from two mixed packages in the applicator apparatus. Finally, we found the empty kidney basins that were used to collect the excess cement intra- and post-operatively weighed 22 g.

**Figure 1:**
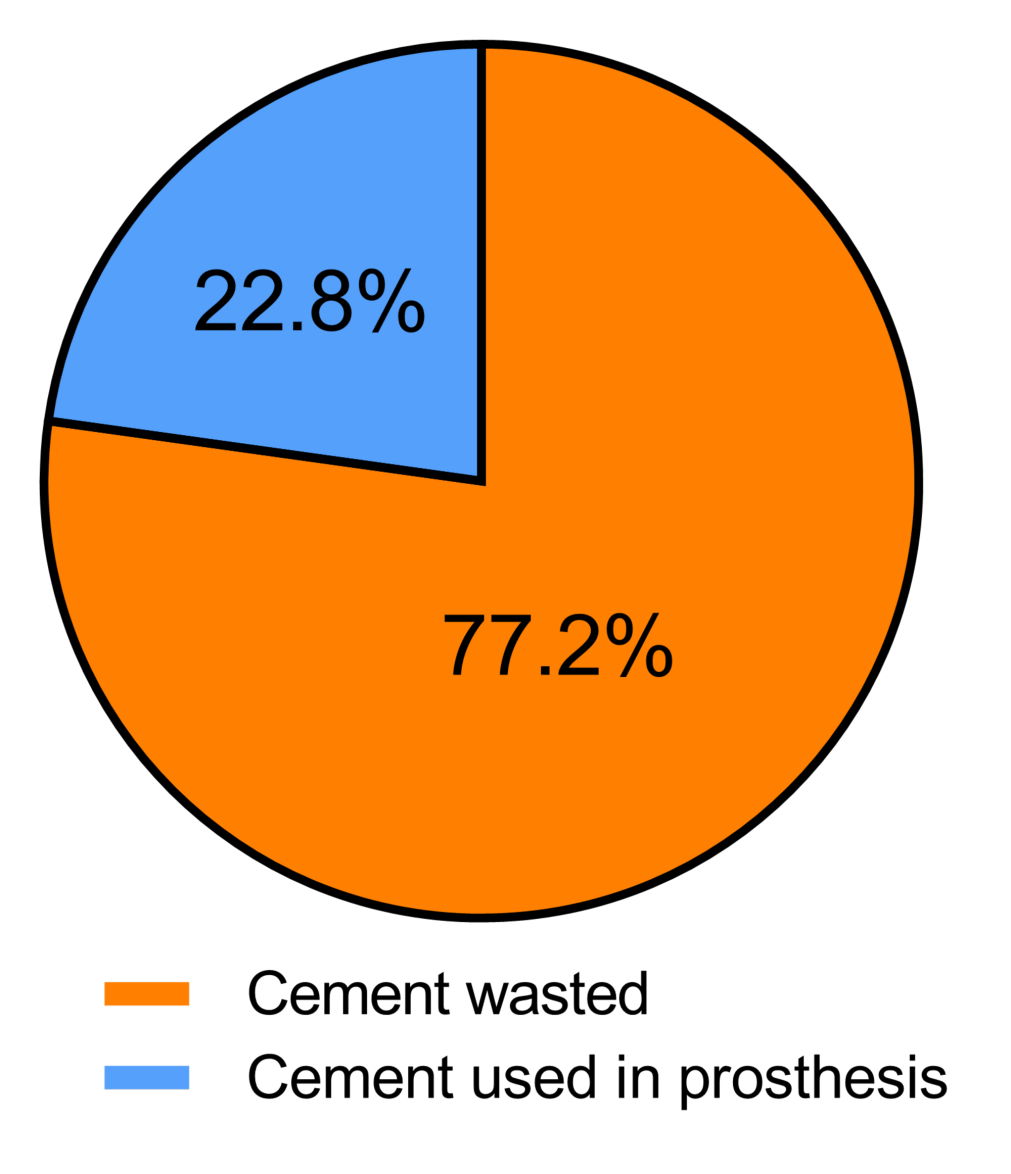
Mean cement waste in total knee arthroplasty (TKA). Cement collected after implantation of components in our series of 102 primary TKAs was considered wasted, this weight was subtracted from the initial total cement used to find the cement remaining in the prosthesis.

Among the 102 patients, the mean weight of the wasted cement collected post-operatively was 91.2 +/- 7.2 g (range 66–107 g). Considering that one prepared package of Simplex® with Tobramycin with the liquid polymer totalled 59 g, this finding implies that less than one package of cement is being retained in the implantation process during the TKA. The mean percentage of cement wasted in a standard primary TKA would be 77.2% by weight (Figure 1). The costs of Simplex® with Tobramycin cement at our institution is $120.62 CAD per package, or $241.24 CAD in cement for a standard case involving two packages. In terms of cost of the amount of cement wasted, 91.2 g represents a wasted value of $186.25 CAD, at a rate of $2.04 CAD per gram of wasted cement.

The relationship between component sizes (i.e., increasing size of tibial base plate, femoral component, and/or resurfacing the patella) and amount of cement wasted was also examined. It was noted that while increasing component size for the tibial (p = 0.07) and femoral implants (p = 0.41) and deciding to resurface the patella (p = 0.14) did result in lower amounts of wasted cement, it was not statistically significant (Table 2).

**Table 2:** Waste according to sizes of arthroplasty component. Average weights of wasted cement per tibial and femoral implant (Stryker Triathlon Knee system) sizes.

Tibial implant sizes	Average weight of wasted cement (g)	Femoral implant sizes	Average weight of wasted cement (g)
2	95.6	2	95.5
3	93.7	3	93.2
4	91.3	4	91.7
5	88.2	5	88.8
6	89.1	6	88
7	86.3	7	89.8
p-value	0.07		0.41

## Discussion

The rising prevalence of TKA will result in an increasing economic challenge for North Americans [1, 2], and hospitals have shown a renewed interest in implementing cost-saving measures within the operating room. Researchers are therefore exploring different avenues for attenuating intraoperative waste accumulation including clinical pathways [6], repurposing of formerly disposable surgical instruments [13, 14], and more recently, reconsidering the amount of bone cement for a standard TKA [15]. As reported [17] by Mayer et al. in a combined systematic literature search and prospective audit of 120 surgeons, antibiotic laden bone cement is becoming increasingly utilized for routine TKAs [18, 19]. Cementing is advantageous in versatility and protection, and the combined antibiotic offers resistance against postoperative infection [19]. Oftentimes excess cement remains after a completed TKA, and although not much is wasted in terms of volume of product, a considerable amount is wasted in terms of cost of product. Maheshwari et al. brought this issue to light by retaining clinical outcomes and implant survivorship and saving approximately $1000 USD per case when using one versus two packets of cement for a standard TKA [15]. Our study, which quantifies exactly how much cement is wasted during a procedure, would be complementary to these findings. As such, the aim of this assessment study was to measure the amount of wasted intraoperative cement for a routine TKA, and to identify a correlation between cement waste and component size. Among 102 prospective primary TKAs, we found the mean weight of excess cement was 91.2 +/- 7.2 g, which is roughly 1.5 times the amount offered by one packet of prepared Simplex® with Tobramycin product. This would indicate that one package of Simplex® with Tobramycin would be sufficient for each TKA procedure. Specifically, considering that a total of 118 g of cement was originally mixed during the operations, then an average of 26.8 g of bone cement was actually retained on the prosthesis. Furthermore, there were no statistically significant correlations found between the amount of cement waste and component size, suggesting that the potential for cost savings is applicable for a wide range of TKA cases.

The present findings are in line with suggestions made by previous literature, which altogether formulate a “less is more” principle concerning bone cement use in primary TKA. As already alluded to, Maheshwari et al. reported no functional deficits in individuals allocated to the one-packet group versus those who underwent a standard TKA with two packets of cement [15]. These investigators also estimated a substantial cost-savings of $523.85 CAD ($430 USD) per case when using one instead of two packets of cement (Simplex® with Tobramycin, 40 g/pack). When applying the mean value of wasted cement found in our study ($186.25 CAD) to the total number of TKAs done in 2016, the economic waste value is an astounding $11,957,995 (CAD). More realistically, the reduced waste will be carried out by the savings of cement packages, and these findings show that only one bag is needed. A saved bag of cement represents unit cost of $120.62 CAD per case, or $7,744,286.48 CAD when applied to 2016 TKA procedure volumes. The potential economic effects of reduced intraoperative cement use are compounded by the fact that regulated waste, such as bone cement, cost hospitals up to 20 times more to process than general waste [20]. Furthermore, Ko et al. performed a prospective analysis of 80 TKA procedures and suggested that a thick cement mantle at the bone-cement interface has negative implications on functional and clinical outcomes [21]. In other words, a conservative approach to cement application might be preferred. Specifically, Nagel et al. [22] and Vanlommel et al. [23] used specimens rather than human subjects to uncover the minimum amount of cement needed to maximize fixation strength during TKA. Their combined evidence proposes a bone-cement penetration depth of no greater than 5 mm during tibial component fixation [22, 23], which is consistent with established standards [24]. Vanlommel et al. reinforced this notion by observing ideal cement penetration of the tibial component with less than 20 g of cement [23], alluding to the minimal amounts of cement actually needed for optimal fixation.

This study has notable limitations. First, the cross-sectional nature of the study prohibits any long-term reporting of implant survivorship or clinical outcomes such as Knee Society assessment or Knee Society function scores. In addition, we were unable to properly perform a multiple regression analysis for our secondary outcomes of component sizes and wasted cement volume. This was likely due to our study being underpowered to get sufficient sample size to make a significant comparison. We did not report on the viscosity of the cement used which might have affected the cementing technique and ultimately the quantity of product used [24]. However, since this study took a pragmatic approach by not deviating from the standard surgical procedure at our institution and including a large and heterogeneous sample population, we can assume each case to have predictable post-TKA outcomes. Finally, our results are influenced by the surgical techniques of our institution’s surgeons as well as limiting the procedure to primary TKAs. We recognize that other surgeons use different methods and may have different supply quantities to achieve similar success in their TKA operations. More complex cases may require more cement as well. However, the utility of this study is to demonstrate that cement waste is contributing to a significant cost lost during TKA procedures.

## Conclusions

We have found that there is considerable waste during a standard TKA procedure in Canada. Particularly, an average of 91 g of cement, or $186.25, is wasted during a standard TKA, which translates to an estimated $12 million CAD of unrealized economic potential over the course of a calendar year. As such, it is our hope that this information will be useful to surgeons who are key stakeholders in promoting and realizing cost reductions in our healthcare system. In addition, this information can help to inform and guide us with respect to the development of more efficient cement delivery systems, collaborating with our equipment suppliers to lobby for different cement aliquant preparations, and may potentially influence surgeons on amount of cement utilized in cemented total knee arthroplasty cases.
